# Supplementary material for: Supervised promoter recognition: a benchmark framework
Source: BMC Bioinformatics. 2022 Apr 2;23:118. doi: 10.1186/s12859-022-04647-5 (PMC8976979; doi:10.1186/s12859-022-04647-5)
Supplement: Supplementary file 1 — Additional file 1. Additional file includes a pdf file with a step-by-step example of our testing framework utilised for DNABERT. [file 12859_2022_4647_MOESM1_ESM.pdf]

Supervised Promoter Recognition: A benchmark framework

SUPPLEMENTARY MATERIALS

This document includes a testing example, including all parameters utilised to reproduced our results. For the re-implementation results, we include multiple bash scripts in the 'previous' folder.

1. TESTING DNABERT

This section will guide you through the replication of our results on our main work for testing a state-of-the-art promoter recognition model known as `DNAbert` [1]. We utilised DNAbert's testing functionality to avoid any inaccuracies that could arise from a re-implementation of their model and be able to use the results obtained as a direct comparison to their presented results.

Before we begin, the repositories we are utilising are the official DNAbert[1] and SUPRREF[2] GitHub repositories, which include readme (README.md) files that provide instructions on their usage. First we clone both repositories and created a 'dnabert environment' as instructed in step 1 (1.1 and 1.2) of their readme file. The NVIDIA Apex installation gave us difficulties and thus we decided to not use fp16, which would not affect us since it is used during training and we are only testing the pre-trained model available from them. Next, we downloaded their model 'DNABERT6', as well as their fine-tuned model for 6 kmers for prom-core. Before we could test the model, we created a dataset utilising `SUPRREF` using the following steps:

> First we installed the command-line tool for suprref as shown in our readme file. Then we created a configuration file with the command 'suprref create configuration'. The previous tool outputs a '.json' configuration file which contains the following parameters:
>
> ```
> {"dataset_configuration": {"sequence_upstream_length": 50,
> "sequence_downstream_length": 250, "stride": 50, "seed": 539606022,
> "error_type": "tss-proximity", "error_margin": [49]}, "input":
> "data/chr1.fa", "annotations": "data/epd_human_tata-all.sga",
> "experiment": "/home/ivan/repos/suprref/test", "dataset": "IO"}
> ```
>
> Next we created the dataset with the configuration file we previously obtained. For this we had to download human chromosome data which can be downloaded using the script found in 'previous/data/human_chrs/download.sh'. Along with this, we must download the TSS from EPD using the command 'suprref download epd -d human -t all'. The dataset creation tool from SUPRREF outputs a '.sld' (sequence label data) file, which we then convert into .tsv (KMER) format required by DNAbert.

Finally, to test DNAbert on our dataset, we followed step 4 in their readme file.

A. Performance

The testing was done in an Azure virtual machine (Standard NC24) with 24 vcpus, 224GiB of memory. The dataset configuration, creation, and conversion took approximately 30 minutes and less than 8GiB of memory. The prediction tool from DNAbert took more than 8 hours and utilised more than 128GiB of memory. Keep in mind that this process did not involve training the model and only covered Human chromosome 1.

REFERENCES

1. Y. Ji, Z. Zhou, H. Liu, and R. V. Davuluri, "DNABERT: pre-trained Bidirectional Encoder Representations from Transformers model for DNA-language in genome," Bioinformatics **37**, 2112–2120 (2021).

---

[1] https://github.com/jerryji1993/DNABERT

[2] https://github.com/ivanpmartell/suprref
